# Genome-wide identification and characterization of ABA receptor PYL/RCAR gene family reveals evolution and roles in drought stress in *Nicotiana tabacum*

**DOI:** 10.1186/s12864-019-5839-2

**Published:** 2019-07-11

**Authors:** Ge Bai, He Xie, Heng Yao, Feng Li, Xuejun Chen, Yihan Zhang, Bingguan Xiao, Jun Yang, Yongping Li, Da-Hai Yang

**Affiliations:** 1 Tobacco Breeding and Biotechnology Research Center, Yunnan Academy of Tobacco Agricultural Sciences, Kunming, Yunnan China; 2 Key Laboratory of Tobacco Biotechnological Breeding, Kunming, Yunnan China; 3 National Tobacco Genetic Engineering Research Center, Kunming, Yunnan China; 4 National Tobacco Gene Research Centre, Zhengzhou Tobacco Research Institute, Zhengzhou, Henan China

**Keywords:** ABA receptor, Drought stress, Gene family, Gene expression, Tetraploid, *Nicotiana tabacum*, *Nicotiana tomentosiformis*, *Nicotiana sylvestris*

## Abstract

**Background:**

Abscisic acid (ABA) is an important phytohormone for plant growth, development and responding to stresses such as drought, salinity, and pathogen infection. Pyrabactin Resistance 1 (PYR1)/PYR1-Like (PYL)/Regulatory Component of ABA Receptor (RCAR) (hereafter referred to as PYLs) has been identified as the ABA receptors. The PYL family members have been well studied in many plants. However, the members of PYL family have not been systematically identified at genome level in cultivated tobacco (*Nicotiana tabacum*) and its two ancestors. In this study, the phylogenic relationships, chromosomal distribution, gene structures, conserved motifs/regions, and expression profiles of *NtPYL*s were analyzed.

**Results:**

We identified 29, 11, 16 *PYL*s in the genomes of allotetraploid *N. tabacum*, and its two diploid ancestors *N. tomentosiformis* and *N. sylvestris*, respectively. The phylogenetic analysis revealed that NtPYLs can be divided into three subfamilies, and each *NtPYL* has one counterpart in *N. sylvestris* or *N. tomentosiformis*. Based on microarray analysis of *NtPYL* transcripts, four *NtPYL*s (from subfamily II, III), and five *NtPYL*s (from subfamily I) are highlighted as potential candidates for further functional characterization in *N. tabacum* seed development, response to ABA, and germination, and resistance to abiotic stresses, respectively. Interestingly, the expression profiles of members in the same *NtPYL* subfamily showed somehow similar patterns in tissues at different developmental stages and in leaves of seedlings under drought stress, suggesting particular NtPYLs might have multiple functions in both plant development and drought stress response.

**Conclusions:**

*NtPYL*s are highlighted for important functions in seed development, germination and response to ABA, and particular in drought tolerance. This work will not only shed light on the PYL family in tobacco, but also provides some valuable information for functional characterization of ABA receptors in *N. tabacum*.

**Electronic supplementary material:**

The online version of this article (10.1186/s12864-019-5839-2) contains supplementary material, which is available to authorized users.

## Background

Abscisic acid (ABA) is an important phytohormone that plays crucial roles in plant growth and development, including seed dormancy and germination, leaf senescence and abscission, bud development, fruit ripening and stomatal aperture control [1–6]. ABA is also a stress indicator for plant in response to stress, such as drought, salinity, and pathogen infection [3, 7–9]. Moreover, many evidences show that there are crosstalks and interactions between ABA and other phytohormones on plant development and responses to environmental cues [3, 10–13].

In plants, ABA is perceived by the ABA receptors to activate ABA signaling cascade. Recently, the Pyrabactin Resistance 1 (PYR1)/PYR1-Like (PYL)/Regulatory Component of ABA Receptor (RCAR) (hereafter referred to as PYLs) were identified as ABA receptors in Arabidopsis through genetic and biochemical screens [14–16]. The ABA-bound PYLs interact with the clade A protein phosphatase type 2Cs (PP2Cs) to prevent PP2Cs from inhibiting of SUCROSE NONFERMENTING 1 (Snf1)-related protein kinase 2s (SnRK2s). Consequently, the liberated SnRK2s could further phosphorylate the downstream substrates to switch on ABA signaling pathway [17–25]. Thus, PYLs, PP2Cs, and SnRK2s consist of the core ABA signaling components [3, 26–29].

Given the important functions of ABA receptors (PYLs) as the core regulators of ABA signaling in plants, many PYLs have been identified/functional characterized in Arabidopsis [8, 14–16, 30–36], rice [37–40], tomato [41, 42], soybean [43], wheat [44], maize [45, 46], poplar [47], rubber tree [48], strawberry [49], cotton [50], and *Brachypodium distachyon* [51, 52]. In the Arabidopsis genome, *AtPYR1* encodes a member of the cyclase subfamily of the START (star-related lipid transfer) domain superfamily, and 13 genes that are similar to *AtPYR1* were named as *AtPYL* (PYR1-like) 1 to *AtPYL*13. Thus, the Arabidopsis PYL family consists of 14 members (AtPYR1 and AtPYL1–13) with a feature of START domain. The 14 AtPYLs have been classified to 3 subfamilies [14, 15, 53]. Moreover, 14 ABA receptor genes were identified from the genome of rubber tree (*Hevea brasiliensis*), and phylogenetic analysis demonstrated that HbPYLs can be divided into three subfamilies [48]. In tomato (*Solanum lycopersicum*), eight *SlPYL*s were isolated, and can be clustered into three subfamilies according to the phylogenetic relationship [54]. In addition, gene structure analysis of the *PYL*s in *H. brasiliensis* and *Brachypodium distachyon* revealed that genomic sequences of *HbPYL*s and *BdPYL*s can be divided into two clades with or without introns [48, 52].

The Static Light Scattering (SLS) and Analytical Ultracentrifugation (AUC) analysis of recombinant AtPYLs in solution revealed that AtPYR1, AtPYL1, and AtPYL2 could form homodimers, while AtPYL4, AtPYL5, AtPYL6, AtPYL8, AtPYL9 and AtPYL10 are monomers [55]. In general, these monomeric AtPYLs interact with AtPP2Cs in an ABA-independent manner, while the dimeric AtPYLs bind to AtPP2Cs in an ABA-dependent manner. The ABA-dependence of AtPYLs-AtPP2Cs interactions are determined by a conserved region which called CL2 [55]. The CL2 region forms an ABA-binding pocket with the other three highly conserved surface loops, CL1, CL3, and CL4 [56]. Therefore, the conserved CL2 region in PYLs is crucial for ABA signaling transduction in plants.

Furthermore, transcription levels of several ABA receptor genes could be regulated in different tissues and by ABA and abiotic stresses. For example, the expression profiles of *Gossypium hirsutum PYL*s were tissue-specific [50]. Twelve *BdPYL*s were identified from the monocot model plant *B. distachyon* genome, and expression levels of *BdPYL11* are significantly down-regulated in response to ABA, NaCl, and osmotic stress [52]. Moreover, expression levels of most *SlPYL*s, except *SlPYL*1 and *SlPYL*8, were also down-regulated by dehydration stress in tomato leaves [54].

Cultivated tobacco (*Nicotiana tabacum*) is not only an important economic crop in many countries, but also widely used as a model for plant biology research. It is well known that the allotetraploid *N. tabacum* (2n = 48, TTSS) was originated from a hybridization event between *N. tomentosiformis* (2n = 24, TT) and *N. sylvestris* (2n = 24, SS) [57, 58]. Therefore, the gene redundancy and diversity of NtPYL family might be more complex than those of AtPYL and OsPYL family in diploid Arabidopsis and rice.

Lots of studies on the ABA receptors have been carried out in many plants, mainly in Arabidopsis [8, 14–16, 30, 31, 36] and rice [37–40]. However, little is known about the ABA receptor family in cultivated tobacco [59]. In this study, we identified 29 *NtPYL*s in the allotetraploid *N. tabacum*, 11 *NtomPYL*s and 16 *NsylPYL*s in the two diploid wild tobacco species *N. tomentosiformis* and *N. sylvestris*, respectively. The phylogenic relationships, chromosomal distribution, gene structures, and the conserved motifs/regions in these *PYL*s were analyzed. Moreover, the expression profiles of *NtPYL*s in tissues at different developmental stages, and in response to drought stress were further investigated. These results provide some valuable information for further functional characterization of ABA receptors in *N. tabacum*.

## Results

### Genome-wide analysis of PYLs in three Nicotiana species

To identify the PYL family members in the *Nicotiana tomentosiformis* (2n = 24, TT), *N. sylvestris* (2n = 24, SS), and *N. tabacum* (2n = 48, TTSS) genomes, the coding sequences and amino acid sequences of 14 Arabidopsis AtPYLs were applied as queries to search against the NCBI database and China Tobacco Genome Database (V2.0) in China Tobacco Gene Research Centre at Zhengzhou Tobacco Research Institute. Based on amino acid sequence similarity to AtPYLs, a total of 11, 16, and 29 *PYL* genes were retrieved from the genomes of two progenitor diploid species *N. tomentosiformis* and *N. sylvestris*, and the descendant tetraploid specie *N. tabacum*, respectively (Additional files 1, 2, 3: Tables S1, S2, S3). As showed in Table 1, the gene and open reading frame (ORF) lengths of *NtPYL*s range from 465 to 3879 bp and 465 to 660 bp, respectively. For the deduced NtPYLs, the protein lengths vary from 154 to 219 amino acids, the molecular weight (MW) from 16.89 to 24.23 kDa, and the Isoelectric Point (pI) from 4.50 to 8.41. Notably, the NtPYL13 and NtPYL22 are the shortest proteins (154 aa) among the NtPYLs.

**Table 1:** Basic information of ABA receptor gene family in *Nicotiana tabacum*

Gene name	Gene ID in database	Gene length (bp)	ORF length (bp)	Deduced Protein		
				Size (aa)	MW (KDa)	pI
*NtPYL 1*	Ntab0128790	726	567	188	21.12	5.1077
*NtPYL 2*	Ntab0298740	1041	570	189	21.21	5.2891
*NtPYL 3*	Ntab0331800	920	561	186	20.90	6.7222
*NtPYL 4*	Ntab0409250	925	564	187	21.08	6.7977
*NtPYL 5*	Ntab0524600	925	564	187	21.08	6.7977
*NtPYL 6*	Ntab0746900	660	660	219	24.23	6.785
*NtPYL 7*	Ntab0790100	657	657	218	24.12	6.5027
*NtPYL 8*	Ntab0830710	630	630	209	23.07	5.917
*NtPYL 9*	Ntab0986250	631	631	210	23.09	5.71
*NtPYL10*	Ntab0025750	648	648	215	23.36	7.5625
*NtPYL11*	Ntab0143100	648	648	215	23.56	7.2493
*NtPYL12*	Ntab0143110	648	648	215	23.56	7.2493
*NtPYL13*	Ntab0350690	465	465	154	16.89	6.5033
*NtPYL14*	Ntab0528630	657	657	218	23.76	8.413
*NtPYL15*	Ntab0012440	642	642	213	23.31	7.137
*NtPYL16*	Ntab0764650	861	642	213	23.34	7.4728
*NtPYL17*	Ntab0430230	636	636	211	23.41	8.19
*NtPYL18*	Ntab0710100	636	636	211	23.34	8.0852
*NtPYL19*	Ntab0177250	2293	528	175	19.59	6.3903
*NtPYL20*	Ntab0568440	1957	561	186	20.75	7.0081
*NtPYL21*	Ntab0424430	2782	561	186	21.09	6.3425
*NtPYL22*	Ntab0906880	1676	465	154	17.32	4.7256
*NtPYL23*	Ntab0217080	2428	561	186	20.84	5.8834
*NtPYL24*	Ntab0010710	2656	564	187	21.34	7.0093
*NtPYL25*	Ntab0725950	3231	564	187	21.37	7.0092
*NtPYL26*	Ntab0504840	3879	534	177	20.16	6.8751
*NtPYL27*	Ntab0868560	3383	534	177	20.23	6.6262
*NtPYL28*	Ntab0282050	691	591	196	21.99	4.5044
*NtPYL29*	Ntab0734960	564	564	187	20.83	4.527

The basic information of the *NtomPYL*s and *NsylPYL*s, including the gene ID, lengths of gene and ORF, MW, and pI are listed in Table 2 and Table 3, respectively. For the *NtomPYL*s, the gene lengths vary from 561 to 3275 bp, and ORF lengths range from 534 to 660 bp. For the deduced NtomPYLs, the protein lengths range from 177 to 219 amino acids, MW from 20.23 to 24.92 kDa, and pI from 4.73 to 7.97 (Table 2). For the *NsylPYL*s, the gene lengths vary from 630 to 3888 bp, and ORF lengths range from 528 to 657 bp. For the deduced NsylPYLs, the protein lengths range from 175 to 218 amino acids, MW from 19.59 to 24.11 kDa, and pI from 4.97 to 8.66 (Table 3). Analysis of the physical properties in the deduced *Nicotiana* PYL members showed that these PYLs are highly conserved. Most of the deduced *Nicotiana* PYLs have the similar amino acid lengths, MW, and theoretical pI. Majority of the putative *Nicotiana* PYLs possess 175–219 amino acids. MW and pI of the deduced *Nicotiana* PYLs range from 16.89 to 25.01 kDa, and 4.50 to 8.66, respectively.

**Table 2:** Basic information of ABA receptor gene family in *Nicotiana tomentosiformis*

Gene ID in database	Gene length (bp)	ORF length (bp)	Deduced Protein		
			Size (aa)	MW(Da)	pI
Ntom0370050	2863	564	187	21.34	7.0093
Ntom0349510	3275	534	177	20.23	6.6262
Ntom0046390	642	642	213	23.31	7.137
Ntom0177100	660	660	219	23.78	7.9746
Ntom0211600	671	660	219	24.23	6.785
Ntom0261490	630	630	209	23.07	5.917
Ntom0128520	648	648	215	23.56	7.2493
Ntom0029000	726	558	185	20.99	5.1022
Ntom0178960	636	636	211	23.34	7.8297
LOC104094947	1049	561	186	20.74	6.44
LOC104091385	561	561	186	20.72	4.73

**Table 3:** Basic information of ABA receptor gene family in *Nicotiana sylvestris*

Gene ID in database	Gene		Deduced Protein		
	length (bp)	ORF length (bp)	Size (aa)	MW (Da)	pI
Nsyl0240040	3230	564	187	21.37	7.0092
Nsyl0402820	3888	534	177	20.18	6.8751
Nsyl0173910	2428	561	186	20.84	5.8834
Nsyl0203250	2295	528	175	19.59	6.3903
Nsyl0129950	1886	537	178	19.99	5.084
Nsyl0376280	657	657	218	24.11	6.5027
Nsyl0312070	630	630	209	23.09	6.1062
Nsyl0435490	642	642	213	23.35	7.4728
Nsyl0104080	648	648	215	23.37	7.5625
Nsyl0463960	636	636	211	23.41	8.0825
Nsyl0370740	925	561	186	20.95	6.7977
Nsyl0089810	1041	570	189	21.21	5.2891
LOC104233805	1399	657	218	23.76	8.66
LOC104247806	707	645	214	24.02	4.97
LOC104234717	1234	561	186	21.10	5.92
LOC104210101	990	561	186	20.95	6.3

### Phylogenetic analysis of PYL family in Arabidopsis, soybean and *N. tabacum*

**Fig. 1:**
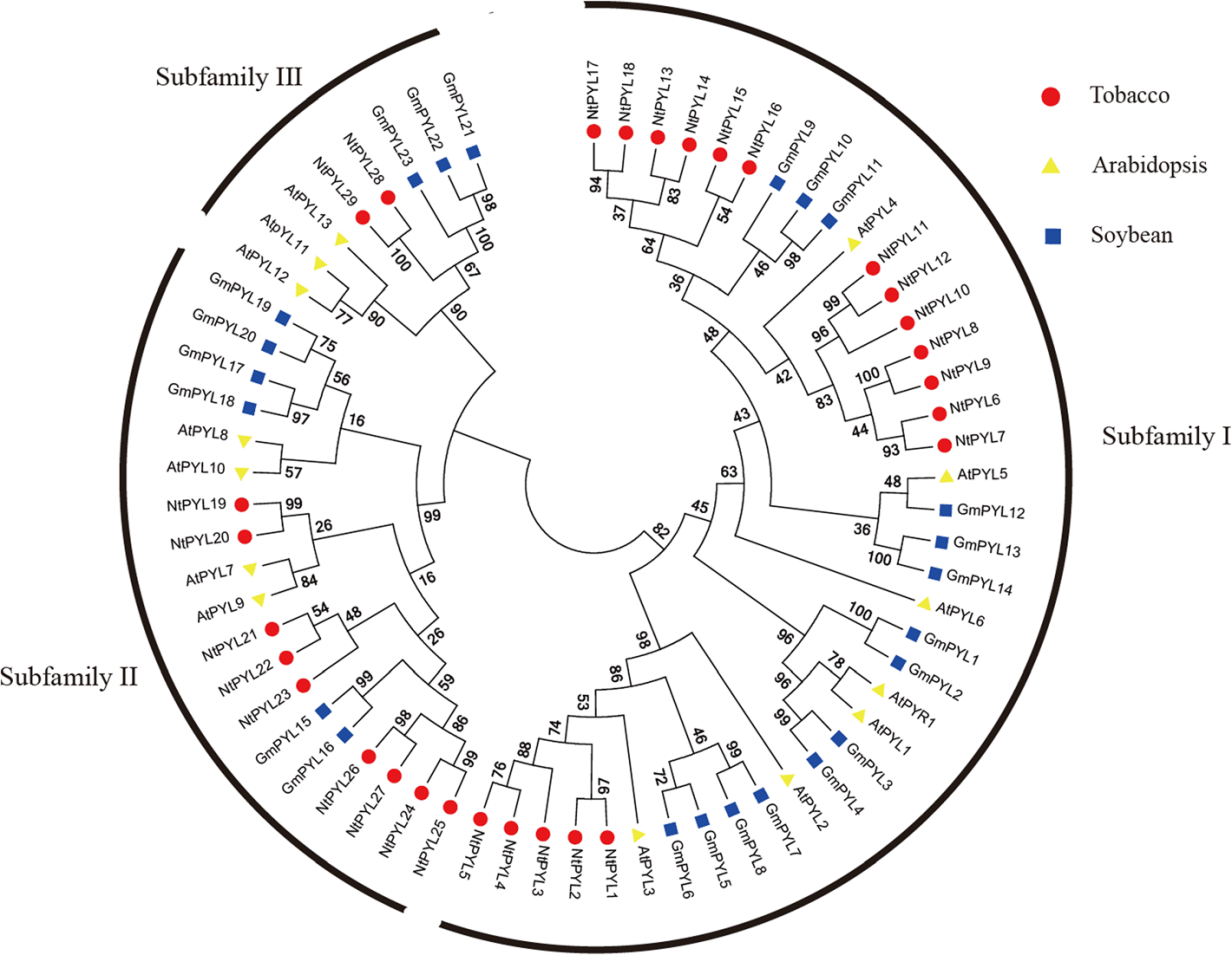
Phylogenetic analysis of PYL proteins from Arabidopsis, soybean and cultivated tobacco. A total of 14 AtPYLs from Arabidopsis (*Arabidopsis thaliana*), 23 GmPYLs from soybean (*Glycine max*), and 29 NtPYLs from cultivated tobacco (*Nicotiana tabacum*) were used to generate the unrooted neighbor-joining (NJ) tree with 1000 bootstrap replicates. The PYL proteins are classified into 3 subfamilies (marked as I, II, III), and distinguished by different colors: AtPYLs labeled in yellow, GmPYLs labeled in blue, and NtPYLs labeled in red

To characterize the phylogenetic relationship among PYLs from Arabidopsis, soybean, and cultivated tobacco, an unrooted neighbor-joining (NJ) tree was constructed using the MEGA software from the alignment of 29 NtPYLs in *N. tabacum*, 14 AtPYLs in *Arabidopsis thaliana*, and 23 GmPYLs in *Glycine max* (Fig. 1). The phylogenetic analysis of amino acid sequences from the deduced NtPYLs, PYLs in Arabidopsis and soybean revealed that the 29 NtPYLs could be grouped with their orthologous PYLs from Arabidopsis and soybean (Fig. 1). Thus, the NtPYLs were renamed as NtPYL1 to NtPYL29 according to their sequence similarities to AtPYLs.

According to the created phylogenetic tree, these PYLs could be classified to 3 subfamilies. Subfamily I include the NtPYL1–18, AtPYR1, AtPYL1 to AtPYL6, and GmPYL1 to GmPYL14. Subfamily II contains NtPYL19 to NtPYL27, AtPYL7 to AtPYL10, and GmPYL15 to GmPYL20. Subfamily III consists of NtPYL28 and NtPYL29, AtPYL11 to AtPYL13, and GmPYL21 to GmPYL23 (Fig. 1). There are 18 NtPYLs, 7 AtPYLs, and 14 GmPYLs in PYL subfamily I; 9 NtPYLs, 4 AtPYLs, and 6 GmPYLs in PYL subfamily II; 2 NtPYLs, 3 AtPYLs, and 3 GmPYLs in PYL subfamily III. Therefore, PYL subfamily I and III have the largest and minimum number of members among the Arabidopsis, soybean, and cultivated tobacco, respectively.

### Chromosomal distributions of NtPYLs

**Fig. 2:**
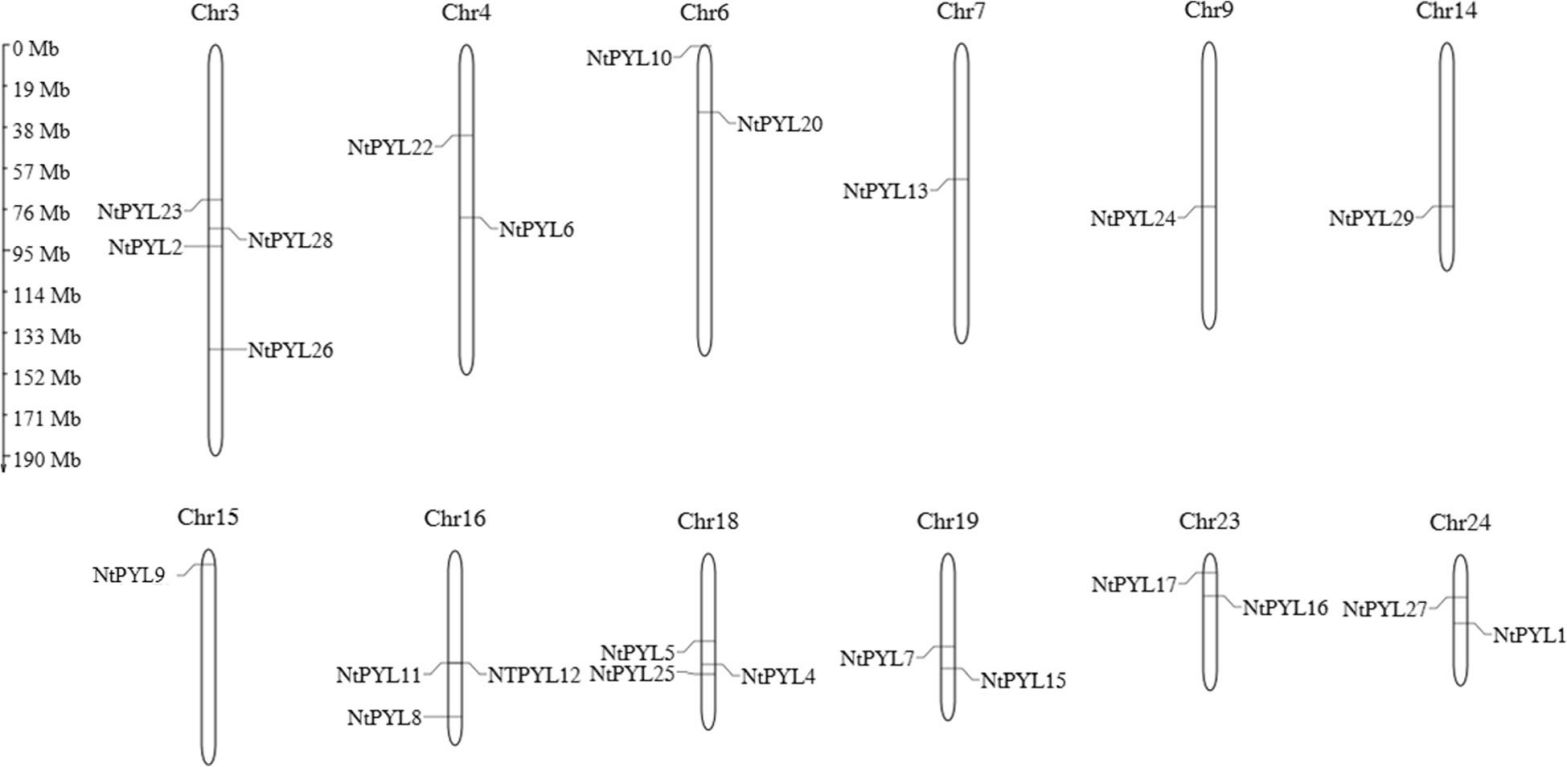
The chromosomal location of the ABA receptor genes (*NtPYL*s) on the *Nicotiana tabacum* chromosomes. Chromosome size is indicated by its relative length. The scale on the left is in megabases (Mb). The bars on the chromosomes indicate the positions of the ABA receptor genes. The figure was generated and modified using the MapGene2Chrom program

The localizations of the PYLs in the chromosomes of *N. tabacum* were further determined. The information of a physical maps (including the length and number of each chromosome, and gene locus) in each *N. tabacum* chromosome were obtained from the China Tobacco Genome Database (V2.0). A simplified physical map which shows the location of *NtPYL*s in the *N. tabacum* chromosomes was drawn by the software MapGene2Chromosome (Fig. 2). In general, *NtPYL*s were unevenly distributed in the *N. tabacum* chromosomes. For example, 24 *NtPYL*s were distributed in the 12 chromosomes, and four *NtPYL*s (*NtPYL*2, *NtPYL*23, *NtPYL*26, and *NtPYL*28) were located on the chromosome 3. Each of chromosome 16 and 18 contains 3 *NtPYL*s (*NtPYL*8, *NtPYL*11, *NtPYL*12 and *NtPYL*4, *NtPYL*5, *NtPYL*25, respectively), while four chromosomes (chromosome 4, 6, 23, and 24) harbor 2 *NtPYL*s, and only 1 *NtPYL* were located on the each of 4 chromosomes (chromosome 7, 9, 14, and 15). However, there are 5 *NtPYL*s that were located on the scaffolds could not be distributed on the *N. tabacum* chromosomes. In addition, *NtomPYL*s and *NsylPYL*s could only be located on the scaffolds but not on the chromosomes of *N. tomentosiformis* and *N. sylvestris*, due to the lacking information of physical maps for the *N. tomentosiformis* and *N. sylvestris*.

Interestingly, according to the analysis of amino acid sequences similarity for PYLs among the *N. tabacum*, *N. tomentosiformis*, and *N. sylvestris*, there should be a tandem gene duplication (*NtPYL11* and *NtPYL*12, both derive from *Ntom0128520*) and gene duplication (*NtPYL4* and *NtPYL5*, both derive from *Nsyl0370740*) event on chromosome 16 and 18, respectively (Fig. 2, Table 4).

**Table 4:** ABA receptor gene family in *Nicotiana tabacum* and its putative ancestors *N. sylvestris* and *N. tomentosiformis*

Gene group	Gene name	Gene ID in database	Orthologous gene
Group 1	*NtPYL1*	Ntab0128790	*Ntom0029000*
	*NtPYL2*	Ntab0298740	Nsyl0089810
Group 2	*NtPYL3*	Ntab0331800	LOC104210101
	*NtPYL4*	Ntab0409250	Nsyl0370740
	*NtPYL5*	Ntab0524600	Nsyl0370740
Group 3	*NtPYL6*	Ntab0746900	*Ntom0211600*
	*NtPYL7*	Ntab0790100	Nsyl0376280
Group 4	*NtPYL8*	Ntab0830710	*Ntom0261490*
	*NtPYL9*	Ntab0986250	Nsyl0312070
Group 5	*NtPYL10*	Ntab0025750	Nsyl0104080
	*NtPYL11*	Ntab0143100	*Ntom0128520*
	*NtPYL12*	Ntab0143110	*Ntom0128520*
Group 6	*NtPYL13*	Ntab0350690	*Ntom0177100*
	*NtPYL14*	Ntab0528630	LOC104233805
Group 7	*NtPYL15*	Ntab0012440	*Ntom0046390*
	*NtPYL16*	Ntab0764650	Nsyl0435490
Group 8	*NtPYL17*	Ntab0430230	Nsyl0463960
	*NtPYL18*	Ntab0710100	*Ntom0178960*
Group 9	*NtPYL19*	Ntab0177250	Nsyl0203250
	*NtPYL20*	Ntab0568440	*LOC104094947*
Group 10	*NtPYL21*	Ntab0424430	LOC104234717
	*NtPYL22*	Ntab0906880	Nsyl0129950
	*NtPYL23*	Ntab0217080	Nsyl0173910
Group 11	*NtPYL24*	Ntab0010710	*Ntom0370050*
	*NtPYL25*	Ntab0725950	Nsyl0240040
Group 12	*NtPYL26*	Ntab0504840	Nsyl0402820
	*NtPYL27*	Ntab0868560	*Ntom0349510*
Group 13	*NtPYL28*	Ntab0282050	LOC104247806
	*NtPYL29*	Ntab0734960	*LOC104091385*

Orthologous genes from *N. tomentosiformis* were labeled in italic

*NtPYL4* and *NtPYL5* in group 2 share the same putative ancestor (marked with underline) from *N. sylvestris*. *NtPYL11* and *NtPYL12* in group 5 share the same putative ancestor (marked with underline) from *N. tomentosiformis*

### Phylogenetic analysis of PYLs in three Nicotiana species

**Fig. 3:**
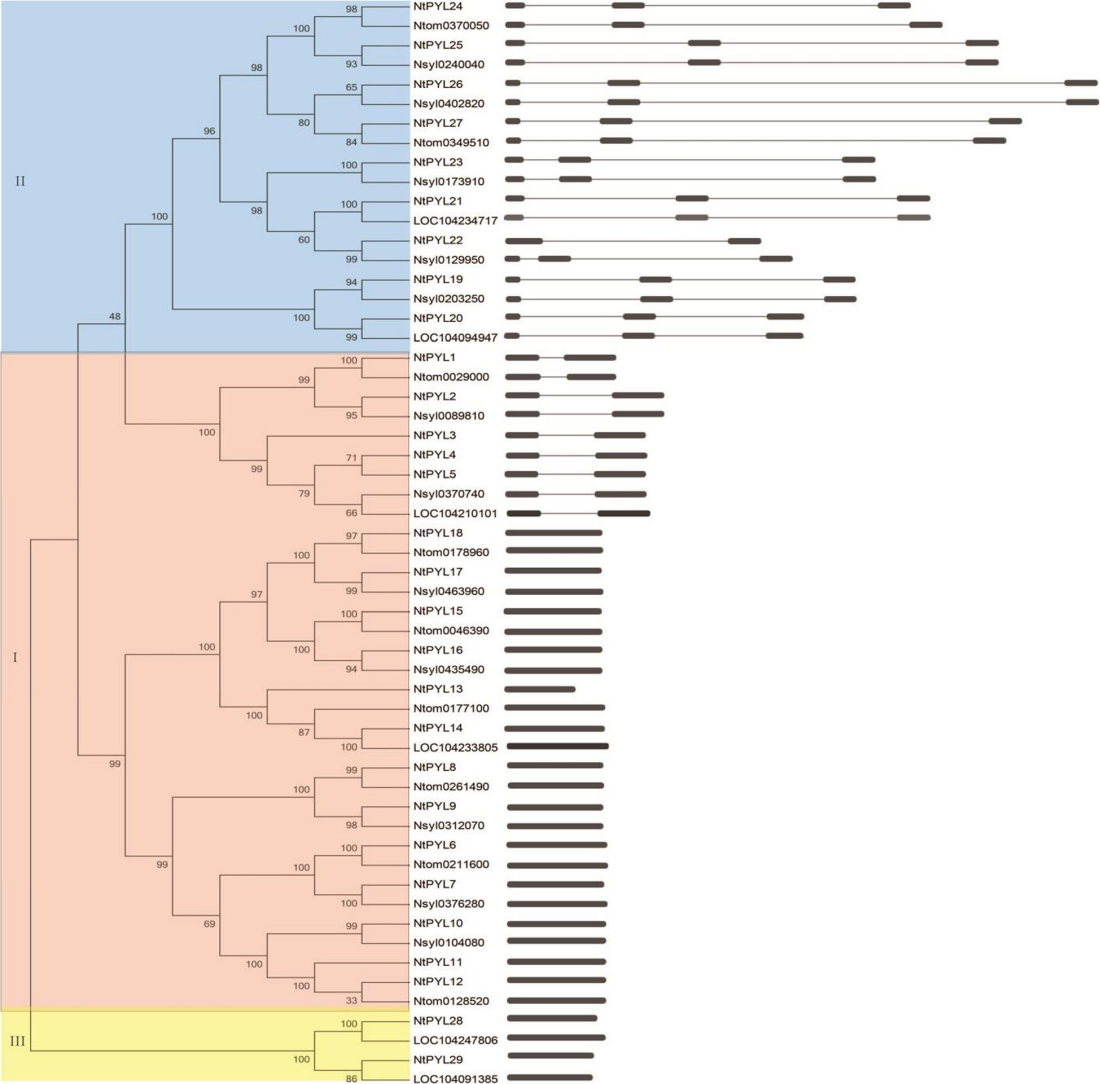
Phylogenetic relationship and gene structure of PYL family in three *Nicotiana* species. The unrooted neighbor-joining (NJ) evolutionary tree was constructed using MEGA 7.0 with 1000 bootstrap replicates based on the deduced full-length amino acid sequences of the PYLs in *Nicotiana tabacum*, *N. tomentosiformis*, and *N. sylvestris* (left panel). Exon-intron analyses of the identical *Nicotiana* PYL genes were performed with GSDS 2.0 (right panel). Exons and introns are represented by black rectangle and black lines, respectively. The lengths of the exons and introns for each *Nicotiana* PYL gene are shown proportionally

Cultivated tobacco (*N. tabacum*) is a tetraploid crop, and the genome of *N. tabacum* (TTSS) is likely derived from two different genomes of two wild diploid tobacco (T genome from *N. tomentosiformis* and S genome from *N. sylvestris*, respectively) [57, 58]. To analyze the phylogenetic relationship among PYLs from *N. tomentosiformis*, *N. sylvestris*, and *N.tabacum*, an unrooted neighbor-joining tree was constructed via the MEGA software by comparing 11 NtomPYLs, 16 NsylPYLs, and 29 NtPYLs (Fig. 3, left panel). The PYLs in these tobacco species could be divided into 3 subfamilies and further classified to 13 groups, and each NtPYL has a putative orthologous gene in either *N. sylvestris* or *N. tomentosiformis* (Fig. 3, Table 4).

Notably, NtPYL4, NtPYL5 in group 2, and NtPYL22, NtPYL23 in group 10 were all derived from *N. sylvestris*, while NtPYL11, NtPYL12 in group 5 are originated from *N. tomentosiformis*. Namely, NtPYL4 and NtPYL5 in group 2 are derived from Nsyl0370740, and NtPYL11 and NtPYL12 in group 5 are derived from Ntom0128520, indicating that NtPYL4 and NtPYL5 might have the same origin from *N. sylvestris*, and NtPYL11 and NtPYL12 might share the same origin from *N. tomentosiformis* (Fig. 3, Table 4).

### Gene structure of PYLs in three Nicotiana species

To better understand the *Nicotiana* PYLs’ structural features, organization of intron/exon was detected via alignment of genomic DNA and ORF sequences of *Nicotiana* PYL family according to the phylogenetic relationship of *Nicotiana* PYLs (Fig. 3, right panel). Fifteen of the 29 *NtPYL*s (*NtPYL*6–18, *NtPYL*28, *NtPYL*29) are intronless, six NtPYLs (*NtPYL*1–5, *NtPYL*22) have one intron, and 8 *NtPYL*s (*NtPYL*19–21, *NtPYL*23–27) have two introns. For the *NtPYL* subfamily I (*NtPYL*1 to *NtPYL*18), five *NtPYL*s (*NtPYL*1 to *NtPYL*5) have one intron, and other 13 members (*NtPYL*6 to *NtPYL*18) does not have intron. Most members in the *NtPYL* subfamily II (*NtPYL*19–27) have two introns, except *NtPYL*22 has only one intron. All members in the *NtPYL* subfamily III (*NtPYL*27 and *NtPYL*28) have no intron (Fig. 3, right panel).

Notably, only *NtPYL*22 in the *NtPYL* subfamily II has one intron, and amino acid length of NtPYL22 (154 aa) is the shortest among the ABA receptors of *N. tabacum* (Table 1). Through comparing the length of exons and introns of members (*NtPYL*19 to *NtPYL*27) in the NtPYL subfamily II, the first exon and intron might had lost in *NtPYL*22 gene.

### Conserved motifs and CL2 regions/loops of NtPYLs

**Fig. 4:**
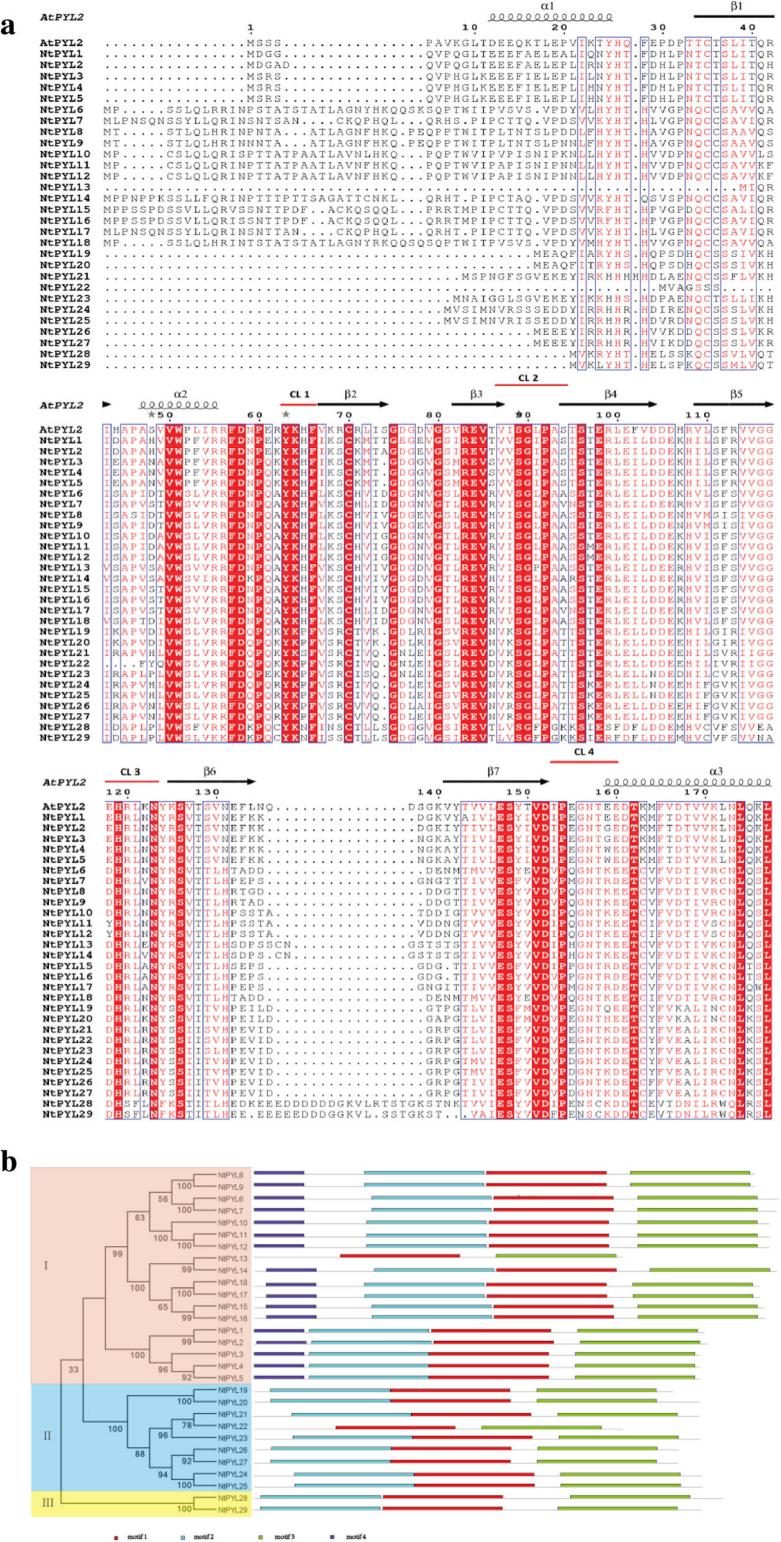
Alignment and conserved motifs of NtPYLs. Amino acid sequence alignment of the 29 NtPYLs and AtPYL2 was performance by ClustalW. **a** Secondary structural elements are indicated above the primary sequence. Helices and sheets/strands are shown as black helices and arrows, respectively. The four conserved ABA receptor region CL1-CL4 are indicated with red lines. The conserved motifs analysis of the NtPYLs based on their phylogenetic relationship were identified using MEME software. **b** In left panel, the members of each subfamily are indicated with the same color and different NtPYL subfamilies are represented by the Roman numeral I-III in the phylogenetic tree. In right panel, grey lines represent non-conserved sequences, and colored boxes numbered at the bottom indicate different motifs. The length of motifs in each NtPYL protein is shown proportionally

Amino acid alignment analysis revealed that all the identified NtPYLs share a highly similar helix-grip organization with three α-helices separated by 7 β-sheets, and several conserved CL regions/loops (Fig. 4a), which have been well characterized in the PYR/PYL/RCAR ABA receptor gene family [14, 15].

The conserved motifs in 29 NtPYLs were predicted via MEME and Pfam software. In total, four motifs were identified in NtPYLs. One motif (motif 4) was identified only in 17 NtPYLs from subfamily I (NtPYL1 to NtPYL18) (Fig. 4b). Most of the 29 NtPYLs share the same motifs (motif 1, 2, and 3) suggesting their conserved functions in the NtPYLs. Among the 29 NtPYLs, only 2 NtPYLs (NtPYL13 from subfamily I and NtPYL22 from subfamily II) have two motifs (motif 1 and 3). Notably, NtPYL13 and NtPYL22 are the shortest receptors (154 aa) in the NtPYL family (Table 1). Moreover, most of the members in subfamily I, except NtPYL13, have the motif 4 at the N-terminal, indicating that most members in NtPYL subfamily I might carry out special biological functions.

**Fig. 5:**
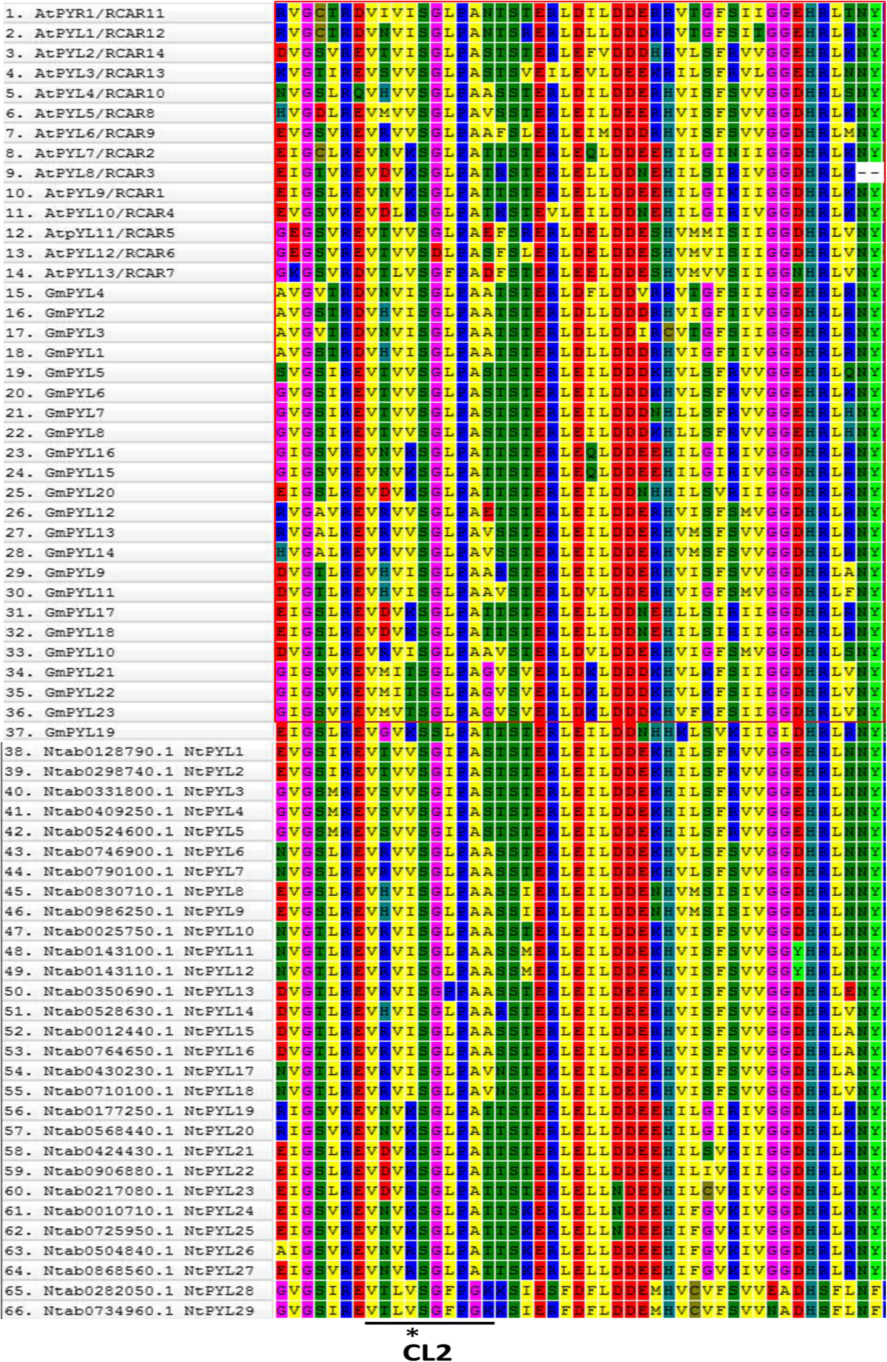
Alignment of the conserved CL2 regions in ABA receptors (PYLs) in Arabidopsis, soybean, and cultivated tobacco. Multiple amino acid sequence alignment of the full-length PYLs from Arabidopsis, soybean, and cultivated tobacco was carried out by ClustalW algorithm. Black line indicates the conserved CL2 loop/region in ABA receptor PYLs; asterisk indicates the position of the two key amino acids

Previous study showed that the conserved CL2 region/loop in PYLs is important for the interaction between PYLs and PP2Cs in an ABA dependent or independent manner in Arabidopsis and soybean [15, 43]. To identify CL2 region in the NtPYLs, alignment of AtPYLs, GmPYLs and NtPYLs was investigated. The conserved CL2 regions including 10 amino acid residues in the NtPYLs show a certain extent of similarity and polymorphism to those in AtPYLs, and GmPYLs (Fig. 5). The No. 3 and 4 residues in CL2 region are the amino acids that are critical for the monomeric or dimeric status, ABA dependence of PYL-PP2C interactions, and activities of AtPYLs [55]. In Arabidopsis, the combination of two key amino acid residues are VI and VV, VK and LK, VV and LV in AtPYL subfamily I, II and III, respectively. In *Glycine max*, the combination of two key amino acid residues are VI and VV, VK, IT and VT in GmPYL subfamily I, II and III, respectively. In *Nicotiana tabacum*, the combination of two key amino acid residues are VI and VV, VK and VR, LV in NtPYL subfamily I, II and III, respectively (Fig. 5).

Common patterns of two key amino acid residues in the CL2 region of AtPYLs, GmPYLs and NtPYLs could be illustrated as followed: VI and VV for subfamily I, VK for subfamily II, and LV is the common pattern in subfamily III of AtPYLs and NtPYLs. The patterns of two key amino acid residues in the CL2 region in AtPYLs, GmPYLs, and NtPYLs are conserved in subfamily I and II among these three plant species, but distinguished in subfamilies III, indicating that the members in different PYL subfamilies might have similar functions in different plants.

### Expression profiles of NtPYLs in tissues at different developmental stages

**Fig. 6:**
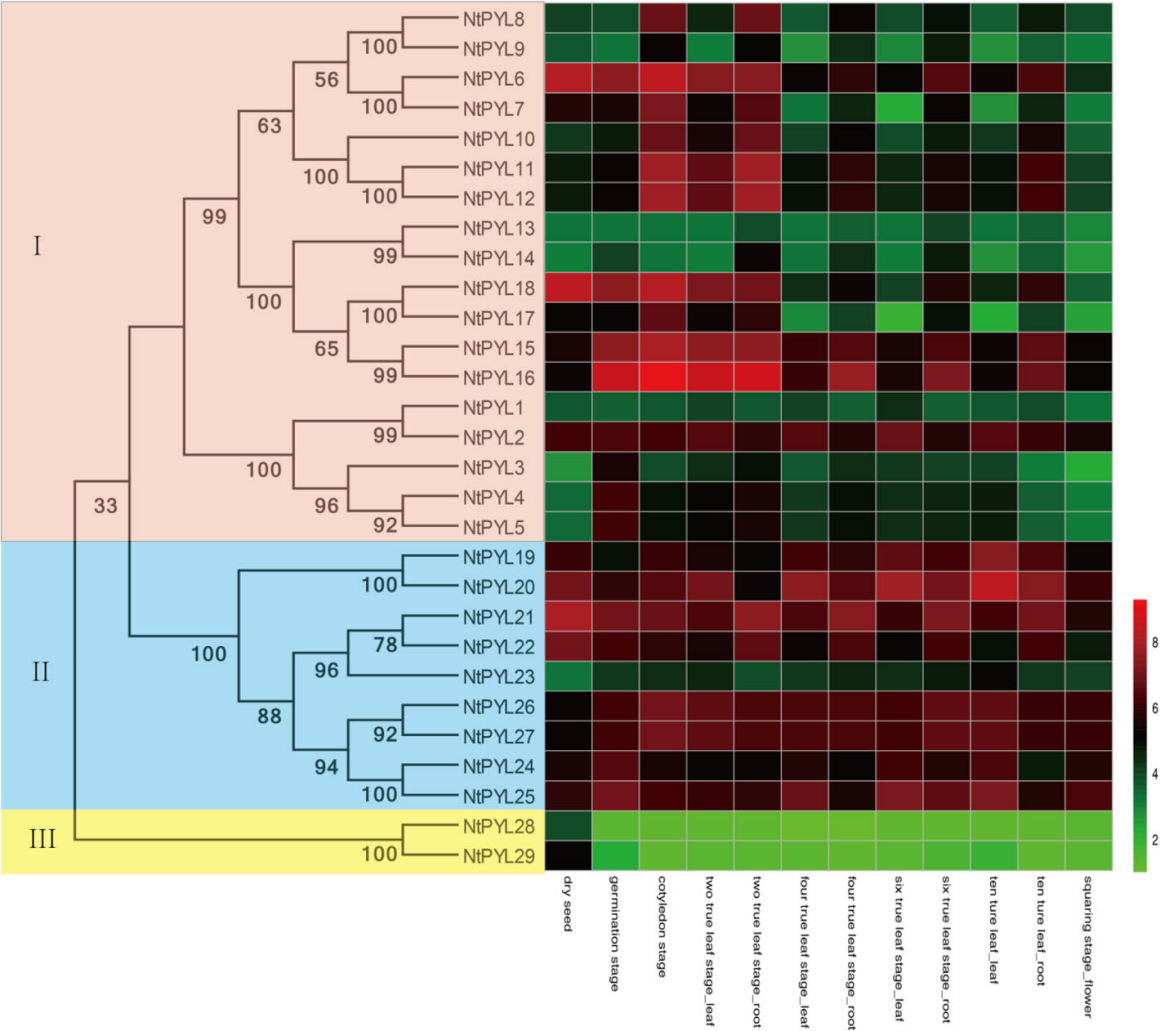
The expression profile of 29 *NtPYL*s in tissues at different developmental stages. The relative transcript abundances of 29 *NtPYL*s were examined via microarray and visualized as a heatmap. The gene expression profiles of *NtPYL*s in 12 different samples, including dry seeds, germination seeds, cotyledons, leaves from two-true leaf stage (labeled as two true leaf_leaf), roots from two-true leaf stage (two true leaf_root), leaves from four-true leaf stage (four true leaf_leaf), roots from four-true leaf stage (four true leaf_root), leaves from six-true leaf stage (six true leaf_leaf), roots from six-true leaf stage (six true leaf_root), leaves from 10-true leaf stage (ten ture leaf_leaf), roots from ten-true leaf stage ten ture leaf_root), and flowers at squaring stage (squaring stage_flower). The X axis is the samples in tissues at different developmental stages. The color scale represents Log2 expression values

To investigate the expression patterns of *NtPYL*s in different tissues and developmental stages, the relative expression levels of *NtPYL*s was analyzed in *N. tabacum* dry seeds, germination seeds, cotyledons, leaves and roots from two, four, six, and ten true leaves stages, and flowers at squaring stage by Microarray analysis (Fig. 6, Additional file 5: Table S5).

The gene expression levels in *NtPYL* subfamily I have a broader range from 2.83 (*NtPYL*3) to 8.33 (*NtPYL*18) in dry seeds. When compared with the expression levels in the dry seeds, expression levels of *NtPYL*3, *NtPYL*4, and *NtPYL*5 were constantly higher in germination seeds, cotyledons, leaves and roots from two, four, and six true leaves stages, and leaves in the ten true leaves stage; expression levels of *NtPYL*10, *NtPYL*11, and *NtPYL*12 were constantly higher in cotyledons, leaves, and roots in two true leaves stage; expression levels of *NtPYL*15 and *NtPYL*16 were higher from germination seeds to roots in the two true leaves stage; expression levels of *NtPYL*6 and *NtPYL*18 were constantly lower from four true leaves stage to flowering stage. Notably, the *NtPYL*7 and *NtPYL*17 had lower expression levels in leaves comparison with roots in the four, six, and ten true leaves stage.

In *NtPYL* subfamily II, the expression levels of most genes (8 of 9 genes) ranged from 5 to 8, except the lower expression level of *NtPYL*23 (3.3) in dry seeds. Compared with the expression levels in the dry seeds, expression level of *NtPYL*23 showed constantly higher in germination seeds till flowers, and expression level of *NtPYL*26 and *NtPYL*27 have constantly higher in cotyledons till leaves in ten true leaves stage. However, *NtPYL*21 and *NtPYL*22 showed lower expression levels in leaves of four, six, and ten true leaves stage and flowers than those in dry seeds. Notably, expression levels of *NtPYL*21 and *NtPYL*22 are lower in leaves compared with roots at four, six, and ten true leaf stages.

For the two members of *NtPYL* family III, *NtPYL*29 (5.13) showed higher expression level than that of *NtPYL*28 (3.84) in dry seeds. Interestingly, expression levels of both *NtPYL*28 and *NtPYL*29 are constantly lower in all the developmental stages after dry seeds stage compared with those in the dry seeds stage (Fig. 6, Additional file 5: Table S5).

### Expression profiles of NtPYLs in response to drought stress

**Fig. 7:**
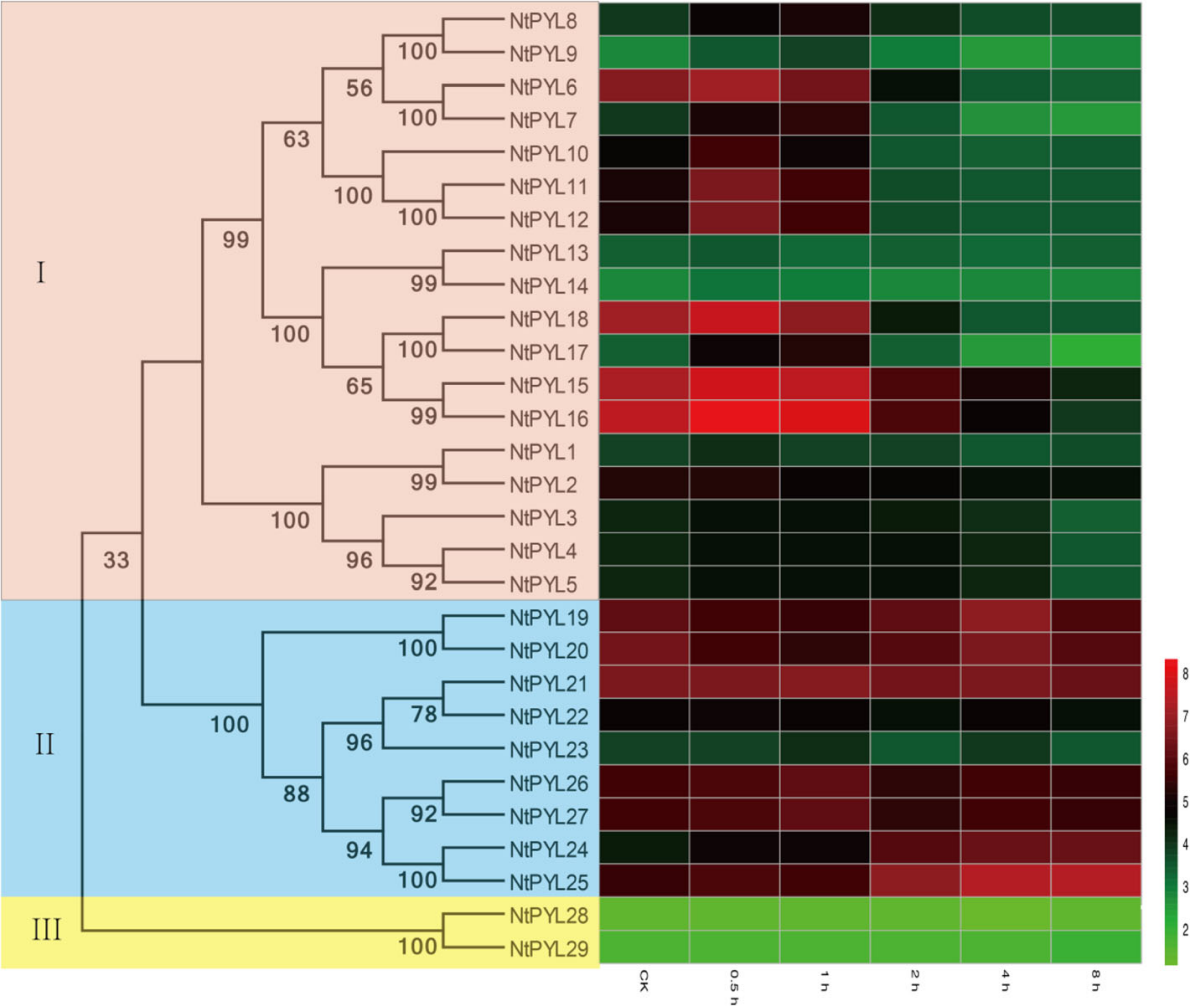
The expression profile of 29 *NtPYL*s in response to drought treatment. The relative transcript abundances of 29 *NtPYL*s were examined via microarray and visualized as a heatmap. The 6–7 weeks old *Nicotiana tabacum* seedings grown in the soil were treated by air dehydration. The X axis is represented for the indicated time after drought treatment. The color scale represents Log2 expression values

To understand the possible function of *NtPYL*s in plant response to drought stress, we analyzed the expression profiles of *NtPYL*s in the tobacco seedlings after drought treatment for indicated time. The expression levels and patterns of all *NtPYL*s were detected by the Microarray. Notably, three pairs of gene, *NtPYL*4 and *NtPYL*5, *NtPYL*11 and *NtPYL*12, and *NtPYL*26 and *NtPY*L27, have identical expression levels and patterns (Fig. 7, Additional file 6: Table S6). The *NtPYL*4 and 5 are derived from *Nsy10370740*, while *NtPYL*11 and 12 are derived from *Ntom0128520* (Fig. 3, Table 4). The amino acid sequence similarity between *NtPYL*26 and 27 is 98.87% (Additional file 4: Table S4). Therefore, the microarray experiment could not distinguish the expression profiles for each of three gene pairs.

In addition, the expression value of each *NtPYL* in CK was assigned as 1, and the expression ratio for the treatment at indicated time/CK was calculated for the expression pattern of each *NtPYL*s (Additional file 6: Table S6). The ratio between treatment and CK that is more than 1.2 or less than 0.8 was recognized as up- or down-regulation, respectively. For the *NtPYL*s in subfamily I, expression levels of 4 *NtPYL*s (*NtPYL*6, *NtPYL*15, *NtPYL*16 and *NtPYL*18) are down-regulated after dehydration treatment for 2 h, expression levels of 7 *NtPYL*s (*NtPYL*7, *NtPYL*8, *NtPYL*9, *NtPYL*10, *NtPYL*11, *NtPYL*12 and *NtPYL*17) are up-regulated after dehydration treatment for 0.5 h and down-regulated thereafter, and expression levels of 7 *NtPYL*s (*NtPYL*1 to *NtPYL*5, *NtPYL*13 and *NtPYL*14) were changed slightly. For the *NtPYL*s in subfamily II, expression levels of 2 *NtPYL*s (*NtPYL*24, 25) are up-regulated after dehydration treatment for 2 h. For the subfamily III, the expression level of *NtPYL*29 is slightly up-regulated after dehydration treatment for 8 h (Fig. 7, Additional file 6: Table S6). Notably, the expression levels of most *NtPYLs* (11 *NtPYLs* in 18 *NtPYLs*) in subfamily I are decreased after dehydration treatment for 2 h, which is consistent with the previous study in tomato [54].

**Fig. 8:**
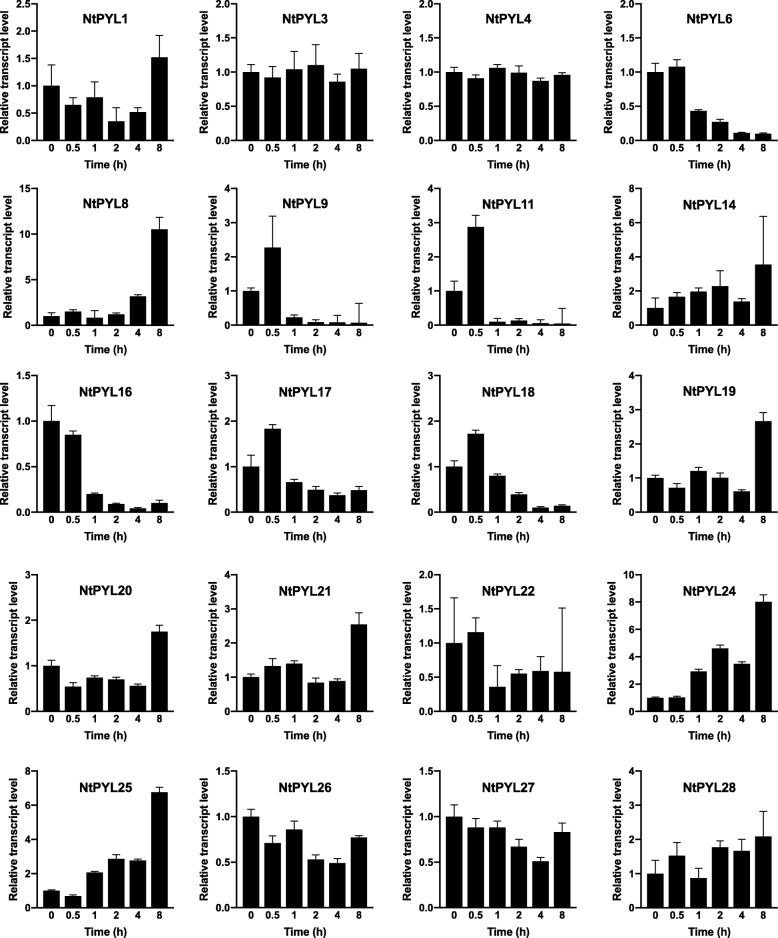
Gene expressions of 20 *NtPYL*s in response to dehydration treatment. The relative transcript levels of 20 *NtPYL*s were examined via quantitative real-time PCR. The 6–7 weeks old *Nicotiana tabacum* seedings grown in the soil were treated by air dehydration

To further confirm the microarray data, gene expression of *NtPYLs* were performed by quantitative real-time PCR. The specific primers of NtPYLs were used for qRT-PCR validation (Additional file 7: Table S7). Consisted with microarray data, gene expressions of *NtPYL*6, *NtPYL16* and *NtPYL18* were down-regulated in response to dehydration treatment, and expression levels of *NtPYL*9, *NtPYL11* and *NtPYL17* were up-regulated for 0.5 h after dehydration treatment. While gene expressions of *NtPYL*1, *NtPYL*3, *NtPYL*4 and *NtPYL*14 were not significantly changed under dehydration treatment (Fig. 8). In addition, transcript levels of *NtPYL24* and *NtPYL25* were up-regulated after dehydration treatment, which is consisted with microarray data (Fig. 8).

## Discussion

Abscisic acid (ABA) is an important phytohormone for plant growth, development, and response to many environmental stresses, particularly in drought stress [1–3, 35]. Recently, genetic and biochemical studies revealed that AtPYLs are ABA receptors in Arabidopsis [14–16], and the ABA receptor PYLs are the key regulators to perceive ABA for initiating ABA signaling [1, 3, 4, 8, 9, 21, 22, 26–28, 60, 61]. PYLs have been characterized in many plants, including Arabidopsis [8, 14–16, 30–36], soybean [43], tomato [41, 42], wheat [44], maize [45, 46], poplar [47], rubber tree [48], cotton [50], and rice [37–40]. However, knowledge about systematical identification and characterization of *Nicotiana* PYL family is very limited at the genome level [59] . In this study, we identified 29, 11, and 16 PYLs based on the similarity to AtPYLs in the genomes of *N. tabacum*, *N. tomentosiformis* and *N. sylvestris*, respectively. The phylogenic relationships, gene structures, chromosomal location, conserved motifs/regions, and expression profiles of *NtPYL* family were analyzed.

According to the coding and amino acid sequences of 14 AtPYLs, 11 NtomPYLs, 16 NsylPYLs, and 29 NtPYLs were retrieved from the genomes of the tetraploid specie *N. tabacum* and its two progenitor diploid species *N. tomentosiformis*, and *N. sylvestris*, respectively. *NtPYL*s, *NtomPYL*s, and *NsylPYL*s and their putative proteins exhibited similar physical properties. In addition, amino acid sequence alignment analysis showed that all the 29 NtPYLs share highly similar structure characterized by several α-helices, β-sheets and conserved CL loops, similar with the AtPYL2, a functional member of the ABA receptor family in Arabidopsis [62]. Therefore, these NtomPYLs, NsylPYLs, and NtPYLs are the ABA receptors in *N. tomentosiformis*, *N. sylvestris*, and *N. tabacum*, respectively. In cotton, PYL families had been identified recently at genome level in two ancestral diploid species *Gossypium raimodii* and *G. arboretum*, and two tetraploid species *G. barbadense* and *G. hirsutum* derived from *G. raimodii* and *G. arboretum*, respectively. Analysis of the physical properties of the 20 GrPYLs, 21 GaPYLs, 39 GrPYLs, and 40 GaPYLs revealed that the *Gossypium* PYL families share similar ORF and amino acid lengths, MW and pI [50]. Compared with the published plant PYL families [14, 15, 38, 41, 48, 50], we found that the *Nicotiana* PYLs had similar physical properties regarding to the amino acid lengths, ORF and MW. Notably, the number of PYL members (29) in *N. tabacum* is larger than those in the tetraploid soybean (23) [43] and other diploid plant species, such as Arabidopsis (14) [14–16], and tomato (15) [41], but lesser than that in the tetraploid cotton (39 or 40) [50], indicating that tetraploid plant species that harbor large PYL families might have more complex ABA responses in plant development and respond to environmental stress.

In Arabidopsis and soybean, *PYL*s could be classified into 3 subfamilies, and each subfamily might have diverse function [14, 15, 43]. The phylogenetic analysis of amino acid sequences of NtPYLs, AtPYLs and GmPYLs showed that NtPYLs could also be grouped into 3 subfamilies. Further analysis also identified 3 subfamilies among three *Nicotiana* species (*N. tomentosiformis*, *N. sylvestris*, and *N. tabacum*). Amino acid sequence alignment of *Nicotiana* PYLs suggested that these PYLs could be further divided into 13 groups (Table 4). The phylogenetic relationships of *Nicotiana* PYLs revealed that each of the 29 NtPYLs had an orthologous ancestor in either *N. tomentosiformis* or *N. sylvestris*. Notably, 16 NsylPYLs and 11 NtomPYLs have been identified in *N. sylvestris* and *N. tomentosiformis*, respectively. These results suggested that both S-genome from *N. sylvestris* (SS) and T-genome from *N. tomentosiformis* (TT) had contributed the PYLs for the genome of *N. tabacum* (TTSS), and S-genome might contribute more PYLs than that of T-genome during evolution [57, 58].

It was showed that *NtPYL*4 and *NtPYL*5 are located in the different positions of chromosome 18 in the *N. tabacum* genome, while *NtPYL11* and *NtPYL12* are almost located in the identical position of *N. tabacum* chromosome 16. These observations suggest that there might be an event of gene duplication for *Nsyl0370740*, the putative ancestor of *NtPYL4* or *NtPYL5,* on *N. tabacum* chromosome 18. Given the physical locations of *NtPYL11* and *NtPYL12* were almost identical on *N. tabacum* chromosome 16, a tandem gene duplication event of *Ntom0128520* might happen during the evolution. However, errors during sequencing, assembling, and/or annotation might result in different annotations for the same gene.

Gene structures of *Nicotiana PYL*s could also provide useful information to understand the evolution relationship of *PYL* family from *N. sylvestris* and *N. tomentosiformis* to *N. tabacum*. For example, since *NtPYL*13, *NtPYL*22 is the shortest *Nicotiana* PYLs (154 aa) identified in this study (Fig. 3, Fig. 4 a), alignment analysis of the gene structures and conserved motifs of NtPYL13, NtPYL22, and their putative ancestors revealed that both NtPYL13 and NtPYL22 are lacking the N-terminal motifs when compared with their putative ancestor Ntom0177100 and Nsyl0129950, respectively (Fig. 4 b). These results indicated that *NtPYL*13 had lost the 5′-region of *Ntom0177100*, and *NtPYL22* had lost the first exon and intron of *Nsyl0129950* during the evolution.

The CL2 loops/regions, particularly the combination of two key amino acid residues in the CL2 region, in AtPYLs determine the ABA-dependence of PYL-PP2C interactions in Arabidopsis [55, 56]. Recent studies on the mutation and degradation of PYLs for PYL-PP2C interactions, together with the downstream substrates of SnRKs, suggest the potential to engineer the core PYLs-PP2Cs-SnRKs components of ABA signaling [30, 32, 63–65]. For the combination of two key amino acid residues in the CL2 region, NtPYLs share the same pattern with those in the AtPYLs, GmPYLs: VI and VV for PYL subfamily I, and VK for PYL subfamily II. AtPYL13, NtPYL28, and NtPYL29 of the PYL subfamily III share the pattern of LV. The conserved combination of two key amino acid residues in the CL2 region in *N. tabacum*, Arabidopsis and soybean, suggest that the CL2 loop/region play vital roles in PYL-PP2C interactions in plants. Whether *N. tabacum* have the similar ABA dependence of PYL-PP2C interactions as those in Arabidopsis deserves further study using yeast two-hybrid and bimolecular fluorescence complementation (BiFC) assays.

Expression profiles analysis in many plants, such as Arabidopsis [5, 35, 36], rice [37, 38, 40], tomato [41, 54], maize [46], cotton [50], and rubber tree [48], revealed that the majority of *PYL*s are expressed in various tissues including seeds, root, leaves, flowers and fruits. For example, most *PYL*s were expressed in all tissues of rice, and *OsPYL*7/8 were highly expressed in embryos, *OsPYL*3 and *OsPYL*5 were primarily expressed in leaves, while *OsPYL*1 was predominantly expressed in roots [38]. In solanaceous tomato, most of the *PYL* genes (including two members of subfamily I, five members of subfamily II, and two members of subfamily III) were highly expressed in roots, while one member (*Sl1g095700*) from *PYL* subfamily III showed the highest expression levels than those of other *PYL*s in leaves. Notably, one gene (*Sl6g061180*) from *PYL* subfamily I and most of the members in *PYL* subfamily II and III exhibited high expression levels during tomato fruit development. Interestingly, transcripts of many fruit-expressed *SlPYL*s gradually increased to the highest level and declined afterwards. Sun et al. also reported that transcripts of *SlPYL1*, *SlPYL2* (two tomato orthologue of AtPYL7 and AtPYL9), *SlPYL3* (a tomato orthologue of AtPYL8 and AtPYL10), and *SlPYL6* (a tomato orthologue of AtPYL4) within the *SlPYL* family fluctuated during fruit development and ripening in tomato [54]. Additionally, expression profile analysis of *PYL*s in a tetraploid cotton (*G. hirsutum*) demonstrated that 22 *GhPYL*s were preferentially expressed in flowers, 10 *GhPYL*s were dominantly expressed in roots, and 3 *GhPYL*s were highly expressed in the fiber [50]. As a tetraploid solanaceous specie, *N. tabacum* might have similar tissue- and developmental stage-specific expression of *PYL*s as those reported in tomato and cotton.

In this study, expression profiles of *NtPYL*s in different tissues and developmental stages were analyzed by chip-Microarray. Transcripts of 29 *NtPYL*s could be detected in all the tissue/developmental stage checked (Fig. 6, Additional file 5: Table S5). Notably, *NtPYL*28 and *NtPYL*29 from subfamily III showed a distinct expression pattern during the development: transcripts of *NtPYL*28 and *NtPYL*29 reached the highest level in dry seeds, reduced dramatically in germination seeds, and remain almost constant low level afterwards. The highly accumulation of *NtPYL*28 and *NtPYL*29 transcripts particularly in dry seeds, suggest that *NtPYL*28 and *NtPYL*29 might play important roles in *N. tabacum* seed dormancy. Kim et al. reported that overexpressing OsPYL/RCAR5 (a rice orthologue of AtPYL8) resulted in hypersensitive to ABA during seed germination and early seedling growth [37]. Overexpressing OsPYL3 (a rice orthologue of AtPYL8) and OsPYL9 (a rice orthologue of AtPYL3) confer ABA hypersensitivity during seed germination in rice [38]. These studies in rice indicated some OsPYLs (OsPYL3, 5, and 9) are involved in seed germination and early seedling growth in response to ABA treatment. Recently, AtPYL8 and AtPYL9 have been shown to play important roles in regulating lateral root growth in the presence of ABA [5, 36]. Given NtPYL19 and NtPYL20 are cluster closely with AtPYL7, AtPYL9, AtPYL8 and AtPYL10 in phylogenetic analysis (Fig. 1), together with the previous studies on functions of the orthologue of AtPYL8 and AtPYL10 in tomato fruit development, and unique expression patterns of *NtPYL*28 and *NtPYL*29 in dry seeds during developmental process, the functions of *NtPYL*19, 20, 28, 29 in seed development, germination, response to ABA deserve further study.

Since the phytohormne ABA play crucial roles in plant responses to drought, we analyzed the expression levels of *NtPYL*s in the whole seedling after dehydration treatment using chip-Microarray (Fig. 7, Additional file 6: Table S6). In general, the transcripts of members in *NtPYL* subfamily II and III remain constant in the control seedlings (CK, without drought stress) and during the drought treatment, indicating that *NtPYL*19–27, *NtPYL*28, and *NtPYL*29 might not involve in the *N. tabacum* seedling responses to drought stress. Interestingly, for members in *NtPYL* subfamily I, the transcriptional responses to drought treatment could be divided into three types: (1) *NtPYL*1–5, 8, 9, 13, and 14 showed constant expression levels; (2) *NtPYL*7, 10–12, and 17 exhibit quick induction (0.5 and 1 h after drought treatment) with a gradually decrease in the later time points (2, 4, and 8 h after drought treatment); (3) *NtPYL*6, 15, 16, and 18 have a constant transcript accumulation in the early time point (0.5 and 1 h after drought treatment) with a clear decline in the later time points (2, 4, and 8 h after drought treatment). The distinct expression patterns of members in *NtPYL* subfamily I suggest that some *NtPYL*s (such as *NtPYL*7, 10–12, and 17) mainly function in the early response to drought stress, while other *NtPYL*s (such as *NtPYL*6, 15, 16, and 18) might play roles in the later response to dehydration.

Overexpression OsPYL/RCAR5 (a rice orthologue of AtPYL8), OsPYL3 (a rice orthologue of AtPYL8), and OsPYL9 (a rice orthologue of AtPYL3) conferred improved drought stress tolerance in rice [37, 38, 40]. Moreover, in leaves of tomato seedlings subjected to dehydration, transcript accumulation of 6 *SlPYL*s (*SlPYL*2–7) were deduced, and expression levels of 3 *SlPYL*s (*SlPYL*4, 6, and 7) were recovered after re-watering [54].

The unexpected results in Arabidopsis showed that expression levels of *AtPYL*s were down-regulated by stress [12]. Overexpression of *AtPYL*5 and *AtPYL*9 resulted in enhanced ABA responses and drought resistance in Arabidopsis through PYL-mediated inhibition of clade A PP2Cs [16, 34, 35]. Importantly, Gonzalez-Guzman et al. revealed that overexpression of tomato monomeric-type, but not dimeric ABA receptors in Arabidopsis confers enhanced resistance to drought stress [41]. In this study, *NtPYL*6–12 in *NtPYL* subfamily I were clustered closely with AtPYL4 and AtPYL5 (Fig. 1), which are monomeric-type ABA receptors [55]. Together with the *NtPYLs* that showed down-regulated expression pattern in *NtPYL* subfamily I, *NtPYL*6, 7, 10, 11, 12 will be the key candidate ABA receptors for functional identification in *N. tabacum* response to drought, salt, and osmotic stresses.

Notably, a comprehensive comparison comparing transcriptional profiles of the core ABA signaling components under osmotic/dehydration stress or ABA treatment between roots and leaves of maize (*Zea mays*) seedlings grew in hydroponic culture revealed that, after treating roots with ABA, the expression of *ZmPYL*s homologous to monomeric-type *AtPYL*s were reduced, whereas those of *ZmPYL*s homologous to dimeric-type *AtPYL*s were increased in maize primary roots. Surprisingly, the opposite pattern was observed in the leaves of the same experiments [46]. This interesting organ-specific expression patterns for ABA receptor genes between roots and leaves of maize in response to ABA suggest that there might be a contrast transcriptional patterns for monomeric- and dimeric-type *NtPYL*s in roots and leaves under abiotic stresses and ABA treatment, which deserves further study in the future study.

Interestingly, the expression patterns of many members in each *NtPYL* subfamily are similar in different tissues/developmental stages and in seedlings under drought stress, suggesting these *NtPYL*s could be regulated by the developmental signals and dehydration treatment as well. Characterization of these NtPYLs is required for understanding their functions during developmental process and in response to stresses.

In the current study, our study identified 29 putative *NtPYL*s in the allotetraploid cultivated tobacco (*N. tabacum*), 11 *NtomPYL*s and 16 *NsylPYL*s in the two diploid ancestral tobacco species, *N. tomentosiformis* and *N. sylvestris*, respectively. We further investigated the physical properties, phylogenetic relationship, protein motifs, and gene structures of *Nicotiana PYL*s. The *Nicotiana* PYLs could also be divided to 3 subfamilies, in consistent with the results from studies in other plants. Phylogenetic, gene structure, and protein motif analysis revealed *NtPYL*s were originated from *NtomPYL*s and *NsylPYL*s, *NtPYL*22 had lost the first exon and intron compared with its origin in *N. sylvestris0129950*, and *NtPYL13* was derived from N-terminal truncation of *N. tomentosiformis0177100* during evolution. Moreover, analysis of *NtPYL*s expression profiles via chip-Microarray assay indicated that each *NtPYL* subfamily might have diverse functions in different tissues/developmental stages and in response to abiotic stresses. Furthermore, four *NtPYL*s (*NtPYL* 19, 20, 28, and 29) in *NtPYL* subfamily II and III were suggested to play important roles in seed development, germination, and response to ABA. Finally, five *NtPYL*s (*NtPYL6,* 7, 10, 11, 12) in *NtPYL* subfamily I were highlighted as potential candidates for further characterizing their functions in *N. tabacum* resistance to abiotic stresses. Taken together, the results from genome-wide identification of *Niacotiana PYL*s will provide some insights on understanding the roles of PYLs in ABA signaling and in response to abiotic stresses in tetraploid plants, which will facilitate the improvement of crop resistance to drought stress.

## Conclusions

ABA receptors (PYLs) play central roles in ABA signaling and plant response to many environmental stresses. Although lots of *PYL* genes and family have been identified in many plant species, the information of *Nicotiana* PYL family is still missing. Here we conducted a genome-wide identification and expression analysis of the PYL family in *N. tabacum*. A total of 29, 11, 16 *Nicotiana PYL* genes were identified in the genome of *N. tabacum* and its two ancestors *N. tomentosiformis* and *N. sylvestris*, respectively. Phylogenetic and gene structure analysis revealed that *Nicotiana* PYL could be divided into 3 subfamilies and 13 groups. Furthermore, each *NtPYL* might have a putative orthologous gene in either *N. sylvestris* or *N. tomentosiformis*, in consistent with the evolutionary origin of *N. tabacum*. The microarray-based analysis of *NtPYL*s expression profiles in tissues at different developmental stages, and in response to drought stress revealed that members in different *NtPYL* subfamily might paly specific roles in *N. tabacum* growth, developmental, and drought stress responses. Interestingly, the expression profiles of members in the same *NtPYL* subfamily showed somehow similar patterns in tissues at different developmental stages and in leaves of seedlings under drought stress, suggesting particular *NtPYL*s might have multiple functions in both plant development and drought stress response. Importantly, four *NtPYL*s (*NtPYL 19*, *NtPYL20*, *NtPYL28*, and *NtPYL29*) are highlighted for the potential functions in seed development, germination and response to ABA. Moreover, five *NtPYL*s (*NtPYL6, NtPYL7*, *NtPYL10*, *NtPYL11*, *NtPYL12*) might play important roles in response to abiotic stresses, particularly in drought. Taken together, these results will facilitate further functional characterization of *NtPYL*s in plant development and in response to abiotic and biotic stresses in tetraploid plants.

## Methods

### Plant materials and growth conditions

The *Nicotiana tabacum* L. cv. Honghuadajingyuan seeds were obtained from Yunnan Academy of Tobacco Agricultural Sciences (Yunnan, China) [66]. Surface-sterilized seeds were directly sowed into the soil in pots. The tobacco young seedlings were grown in the plant growth chamber with a 16-h-light/8-h-dark photoperiod under continuous white light (∼75 mol m^− 2^ s^− 1^) at 28 °C-day/ 23 °C-night. All plants were kept well-watered after sowing.

For expression profiling of ABA receptor genes in response to drought stress, the plants were grown for 7–8 weeks with 6–7 leaves. The plants were moved out from the pots carefully without disturbing the root, and the surface soil was washed out softly. Then the plants were put on the bench for air drying which termed as drought stress treatment. The whole seedlings were collected at the indicated time after treatment, and immediately frozen in liquid nitrogen for RNA extraction for microarray assay. Five biological replicates were used for sample harvesting at each indicated time of the treatment.

For expression profiling of ABA receptor genes in different developmental stages and tissues in tobacco, tobacco plants were kept in the growing condition mentioned above. Samples were harvested from 12 tissues at different developmental stages, including dry seed, germination seeds, cotyledon, leaves and roots from two, four, six, and ten true-leaf stage, respectively, and flowers at squaring stage. Samples were immediately frozen in liquid nitrogen for RNA extraction for microarray assay. Three to five biological replicates were used for sample harvesting.

### Analyses of phylogenetic, conserved motif, isoelectric point prediction, gene structure, and chromosome localization

The *Nicotiana* PYL gene sequences were retrieved from NCBI (https://www.ncbi.nlm.nih.gov/) and China tobacco genome database V2.0 (data not published). The genomic DNA, open reading frame, and deduced protein sequences of *Nicotiana* PYL family are provided in the (Additional files 1, 2, 3: Tables S1, S2, S3), and had been submitted to NCBI database (currently waiting for assigning the accession numbers).

The sequences of GmPYLs and AtPYLs were retrieved from the NCBI GeneBank database. The sequences of NtPYLs, AtPYLs, and GmPYLs were aligned using ClustalW [67], and an unrooted phylogenetic tree was generated using MEGA 7.0 software (http://www.megasoftware.net) by the neighbor-joining method with 1000 replicates of bootstrap analysis. For analyzing secondary structure of NtPYLs, the above alignment results were further treated by ESPript 3.0 software with default parameter settings (http://espript.ibcp.fr/ESPript/cgi-bin/ESPript.cgi).

Protein motifs were predicted by motif elicitation program MEME (http://meme-suite.org/tools/meme). The conserved motifs were further queried in the InterPro database (http://www.ebi.ac.uk/interpro). The isoelectric point and molecular weight of deduced NtPYL proteins were predicted by ProtParam tool (http://web.expasy.org/protparam/).

For gene structure analysis, the open reading frame sequence of each NtPYL was aligned with its corresponding genomic DNA sequence to identify the gene structure by GSDS 2.0 (http://gsds.cbi.pku.edu.cn/). The chromosome localization of *NtPYL*s were mapped with localization MapGene2Chromosome V2 (http://mg2c.iask.in/mg2c_v2.0/).

### Transcriptomic microarray analysis

Total RNA was extracted with SuperPure Plantpoly RNA Kit (GeneAnswer, China). All RNA samples were treated with RNase-free DNase I (GeneAnswer) and analyzed for integrity on a Bioanalyzer 2100 (Agilent technologies, USA). About 33.3 ng total RNA were used for amplification with WT Amplification Kit (Affymetrix, Thermo Fisher Scientific, USA). 5.5 μg of the amplified product were fragmented by uracil-DNA glycosylase and apurinic/apyrimidinic endonuclease 1 (Affymetrix, Thermo Fisher Scientific, USA).

The fragmented cDNA was labeled by terminal deoxynucleotidyltransferase using the DNA Labeling Reagent (Affymetrix, Thermo Fisher Scientific, USA) that was covalently linked to biotin. The resulting labeled cDNAs (5.2 μg) were dissolved in 160 μl of hybridization mix solution, then denatured at 99 °C for 5 min. The mixed hybridization buffer was loaded into a microarray, and then the both septa were covered by round labels to prevent leaks and evaporation.

An Affymetrix custom Tobacco Genome Array with feature Size 5 μm was used. Eighty thousand six hundred fifty-two tobacco genes were covered within this array. Tobacco L25, EF1-alpha, Ntubc2, PP2A genes were used as housekeeping genes. RMA method provided by the R package, affy package, was used to conduct background correction, normalization, probe-specific background correction, probe summarization and to convert probe level data to expression values.

The hybridizations were performed in a hybridization oven (Affymetrix, Thermo Fisher Scientific, USA) at 45 °C for 16 h. After hybridization, microarrays were washed by Fluidics Station 450 with wash buffer A and B (Affymetrix, Thermo Fisher Scientific, USA). Three biological replicates were used in the Microarrays assay. The expression levels of members in *NtPYL* family in several tissues at different developmental stages and in response to drought stress were documented in Additional file 5: Table S5 and Additional file 6: Table S6, respectively.

### qRT-PCR validation of chip-microarray

For RT-qPCR validation of expression pattern of ABA receptor genes in response to drought stress, the plants were grown for 7–8 weeks with 6–7 leaves. The plants were moved out from the pots carefully without disturbing the root, and the surface soil was washed out softly. Then the plants were put on the bench for air drying which termed as drought stress treatment. The whole shoots were collected at the indicated time after treatment, and immediately frozen in liquid nitrogen for RNA extraction. Three to five biological replicates were used for sample harvesting at each indicated time of the treatment.

RNA was extracted from three to five biological replicates of the whole shoots collected at the indicated time after drought treatment using the Qiagen RNeasy Plant Mini Kit (Qiagen, Hilden, Germany) following the manufacturer instructions.

2 μg of total RNA in a 20 μl reaction was converted to cDNA with a SuperScript III Reverse Transcriptase (Invitrogen, USA) by manufacturer instructions on a Eppendorf Mastercycler thermocycler (Eppendorf AG, Germany) with the following conditions: 25 °C for 5 min, 50 °C for 60 min, 70 °C for 15 min, followed by a hold at 4 °C until use in RT-qPCR reaction. 60 μl deionized water was added into 20 μl cDNA, and 1 μl of diluted cDNA mixture was used as the input for qPCR reaction. qPCR reactions were made with a SuperReal PreMix Plus SYBR Green Kit (TIANGEN Biotech, China) following manufacturer instructions in a 20 μl volume. The specific primers of NtPYLs were used for qRT-PCR validation (Additional file 7: Table S7).

qPCR was done on an Applied Biosystems™ QuantStudio™ 6 Flex Real-Time PCR System (ThemoFisher Scientific, USA) with the following cycling conditions: 95 °C for 15 min, followed by 40 cycles of 95 °C for 10 s, 60 °C for 20 s, and 72 °C for 32 s. Melt curve conditions were 95 °C for 15 s, 60 °C for 1 min, 95 °C for 15 s. All samples had only one melt temperature peak. Fold change between experimental samples (with drought treatment) and control samples (without drought treatment) was calculated by the 2^-ΔΔCT^ method using 26S as a reference gene. CT values represent the average of three technical replicates.

### Statistical analysis

The presented values are the means ± SE of three individual experiments with three replicated measurements. An analysis of variance (ANOVA) was used to compare significant differences based on significance levels of *P* < 0.05 and *P* < 0.01.

## Additional files

Additional file 1: **Table S1**. Genomic DNA sequences of *NtPYL*s, *NtomPYL*s, *NsylPYL*s. (XLSX 34 kb)

Additional file 2: **Table S2**. Coding sequences of *NtPYL*s, *NtomPYL*s, *NsylPYL*s. (XLSX 19 kb)

Additional file 3: **Table S3**. Deduced amino acid sequences of NtPYLs, NtomPYLs, NsylPYLs. (XLSX 14 kb)

Additional file 4: **Table S4**. Matrix of amino acid sequence similarity of NtPYLs. (XLSX 16 kb)

Additional file 5: **Table S5**. Expression levels of *NtPYL*s in tissues at different developmental stages. (XLSX 37 kb)

Additional file 6: **Table S6**. Expression levels of *NtPYL*s in response to drought treatment. (XLSX 22 kb)

Additional file 7: **Table S7**. List of primers used for qRT-PCR validation. (XLSX 10 kb)

## Data Availability

The original data that support the findings of this study are available from National Tobacco Gene Research Centre at Zhengzhou Tobacco Research Institute but restrictions apply to the availability of these data, which were used under license for the current study, and so are not publicly available. Data are however available from the authors upon reasonable request and with permission of National Tobacco Gene Research Centre at Zhengzhou Tobacco Research Institute.

## Abbreviations

ABA: Abscisic acid
MW: Molecular weight
NJ: Neighbor-joining
ORF: Open reading frame
pI: isoelectric point
PYL: PYR1-Like
PYR: Pyrabactin Resistance
RCAR: Regulatory Component of ABA Receptor
